# Unveiling the Mystery of Adult-Onset Still’s Disease: A Compelling Case Report

**DOI:** 10.3390/life14020195

**Published:** 2024-01-29

**Authors:** Daniele Sola, Carlo Smirne, Francesco Bruggi, Chiara Bottino Sbaratta, Aubin Cardin Tamen Njata, Guido Valente, Maria Cristina Pavanelli, Rosetta Vitetta, Mattia Bellan, Lorenzo De Paoli, Mario Pirisi

**Affiliations:** 1 Department of Translational Medicine, Università del Piemonte Orientale, 28100 Novara, Italy; carlo.smirne@med.uniupo.it (C.S.); f.bruggi@gmail.com (F.B.); chiarabottinosbaratta@gmail.com (C.B.S.); cardin52000@yahoo.fr (A.C.T.N.); guido.valente@med.uniupo.it (G.V.); mattia.bellan@med.uniupo.it (M.B.); mario.pirisi@med.uniupo.it (M.P.); 2 Internal Medicine Unit, Maggiore della Carità Hospital, 28100 Novara, Italy; 3 CAAD (Center for Autoimmune and Allergic Diseases), Università del Piemonte Orientale, 28100 Novara, Italy; 4 IRCAD (Interdisciplinary Research Center of Autoimmune Diseases), Università del Piemonte Orientale, 28100 Novara, Italy; 5 Pathology Unit, Sant’Andrea Hospital, 13100 Vercelli, Italy; cristina.pavanelli@aslvc.piemonte.it; 6 Rheumatology Unit, Sant’Andrea Hospital, 13100 Vercelli, Italy; rosetta.vitetta@aslvc.piemonte.it; 7 Hematology Unit, Sant’Andrea Hospital, 13100 Vercelli, Italy; lorenzo.depaoli@aslvc.piemonte.it

**Keywords:** adult-onset Still’s disease, macrophage activation syndrome, hemophagocytic lymphohistiocytosis, autoimmunity, autoinflammatory diseases, immune system, inflammation, steroids, fever of unknown origin, monocytes/macrophages

## Abstract

Adult-onset Still’s disease (AOSD) is a rare systemic inflammatory disorder. Diagnosis can take a long time, especially in the presence of confounding factors, and it is, to some extent, a process of exclusion. AOSD has life-threating complications ranging from asymptomatic to severe, such as macrophage activation syndrome (MAS), which is also referred to as hemophagocytic lymphohistocytosis (HLH). This condition is correlated with cytokine storm production and monocyte/macrophage overactivation and typically occurs with rash, pyrexia, pancytopenia, hepatosplenomegaly and systemic involvement. Exitus occurs in approximately 10% of cases. For the treatment of MAS-HLH, the Histiocyte Society currently suggests high-dose corticosteroids, with the possible addition of cyclosporine A, anti-interleukin (IL)-1, or IL-6 biological drugs; the inclusion of etoposide is recommended for the most severe conditions. In all cases, a multidisciplinary collaboration involving the resources and expertise of several specialists (e.g., rheumatologist, infectiologist, critical care medicine specialist) is advised. Herein, we provide a detailed description of the clinical case of a previously healthy young woman in which MAS developed as a dramatic onset manifestation of AOSD and whose diagnosis posed a real clinical challenge; the condition was finally resolved by applying the HLH-94 protocol (i.e., etoposide in combination with dexamethasone).

## 1. Introduction

Adult-onset Still’s disease (AOSD) is a complex autoimmune inflammatory disease of unknown etiology. It is a rare condition (annual incidence of 0.16 per 1,000,000 people), with a variable spectrum of signs/symptoms at onset which can mimic other inflammatory or infectious conditions [1]. AODS usually affects young people, without gender preference, and presents with two peaks at 20 and 40 years of age, even if elderly cases have been described in some cohorts [2]. Diagnosis is not always easy; it is often delayed due to clinical heterogeneity and involves a process of exclusion. The diagnostic process can be guided by Yamaguchi (Table S1) [3] or Fautrel criteria (Table S2) [4].

Looking more closely at the aforementioned classifications, it can be seen that the clinical elements of AOSD include signs/symptoms (such as fever, arthralgia, rash, pharyngodynia, splenomegaly, lymphadenopathy) and alterations in laboratory tests (such as leukocytosis, neutrophilia, increased hepatic cytolysis indices) that are shared with numerous other rheumatological, infectious, or neoplastic conditions which should be reasonably excluded. The problem is that these algorithms can assist in the diagnostic process only when it has already been considered by the treating physician.

AOSD typically presents in a mild and self-limiting way, but, in some cases, it may exhibit a more tumultuous onset or rapidly progress to fearsome complications. An uncommon yet severe evolution of AOSD is macrophage activation syndrome (MAS), a form of hemophagocytic lymphohistiocytosis (HLH) occurring within immunorheumatological diseases. As reported by some retrospective studies, between 6 and 19% of AOSD cases evolve into MAS [5,6]. MAS can occur at the time of diagnosis or, more rarely, during the disease course [7]. The decisive diagnostic factor is biopsy, with special reference to the presence of differentiated histiocytes engaged in the phagocytosis of red series hematopoietic elements. Clinically, suspicion of HLH should arise from constitutional symptoms, with or without high fever, and some not-so-specific laboratory abnormalities. The latter ones include anemia with thrombocytopenia/leukopenia, a significant gap between elevated ferritin and a lower increased erythrocyte sedimentation rate, and high triglyceride levels with normal or low blood fibrinogen (Table S3) [8,9,10,11].

The prognosis of MAS varies and strictly depends on the diagnostic delay. Early recognition and mild cases often lead to a rapid resolution. However, conditions with poor prognosis and fatal outcomes have also been reported. Therefore, MAS should always be considered a serious manifestation requiring aggressive treatment [12]. The Histiocyte Society has recommended the use of etoposide (VP-16) combined with dexamethasone for treating various forms of HLH, including the autoimmune-related one, since 1994 [8,13,14,15]. A valid alternative is represented by anakinra (a recombinant human interleukin (IL)-1 receptor antagonist), especially via the intravenous route (4 times a day) interspersed with boluses of high-dose methylprednisolone [16]. Useful drugs in this context, alone or in combination, also include tocilizumab (an anti-IL-6 receptor monoclonal antibody), cyclophosphamide, intravenous immunoglobulin (IVIG), and emapalumab (an anti-interferon gamma monoclonal antibody) [7]. Optimal management always includes adequate prophylaxis for bacterial and viral infections and the prevention of osteoporosis if corticosteroids (CSs) are part of the therapeutical regimen.

Here, we report a case of MAS secondary to AOSD (hereafter referred to as AOSD-MAS) in a previously healthy patient who responded to the aforementioned therapies with CS and etoposide. In our opinion, this is a paradigmatic example of how coexisting medical diagnoses may condition the judgment of the treating physicians, possibly leading to potentially very dangerous underestimations of problems. This case is, therefore, suitable for educational purposes for doctors who may not have a specialization or at least a specific interest in the care of rheumatological patients.

## 2. Case Presentation

**Figure 1:**
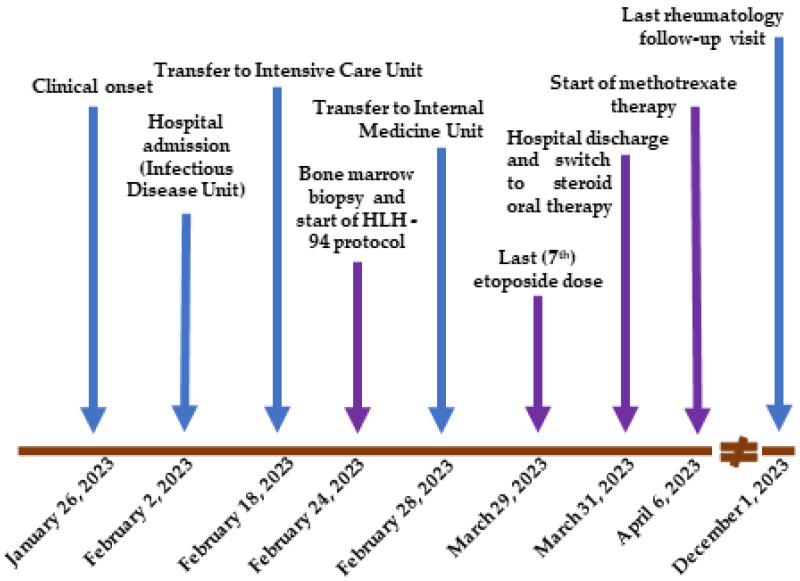
Timeline with relevant data from the clinical history.

A 34-year-old woman with a history of cigarette smoking and unspecified bronchial asthma in childhood which resolved spontaneously during school age presented to the Emergency Department for persistent fever over 39 °C lasting for one week and associated with general malaise, symmetrical arthralgias localized to the knees, wrists, and ankles, pharyngodynia, and cervical lymphadenopathy. Four days before hospital admission, the patient started a home therapy with amoxicillin 1 g three times a day (t.i.d) and non-steroidal anti-inflammatory drugs (NSAIDs) but obtained an inadequate control of body temperature, which never fell below 38.5 °C. She also reported headaches and skin rashes, which tended to be migratory and transient. The complete timeline of her clinical case is presented in Figure 1.

The patient was admitted to the Infectious Diseases Department. At clinical examination, she was severely ill and febrile, with physical weakness and anorexia as the main associated constitutional symptoms; she complained of headache and diffuse arthralgias. Salmon-pink nonpruritic skin rashes were evident on the trunk and extremities during fever episodes; cervical lymph nodes, liver, and spleen were palpable. Data from the main initial blood tests, showing a severe inflammatory state, are presented in Table 1.

Serial blood cultures were obtained, of which only one was found to be positive for meticillin-sensitive Streptococcus Thermophilus. Targeted antibiotic therapy with ceftriaxone 2 g quaque die (q.d.) and teicoplanin (maintenance dose: 12 mg/kg q.d.) was then started and discontinued after five days both due to lack of clinical response and the development of diarrhea. A transesophageal echocardiography was performed and showed no endocardial vegetations. As stool samples were positive for Clostridium difficile, the patient also received targeted antibiotic therapy with oral vancomycin 500 mg q.d.

After her hospital admission, the patient’s clinical condition continued to deteriorate with hemodynamic instability (systolic pressure of 75 mmHg during crystalloid fluid replacement), so after two weeks she was transferred to the Intensive Care Unit (ICU) with a working diagnosis of “fever of unknown origin associated with severe hypotension”. In the ICU, vasopressin and norepinephrine were administered to support organ perfusion. Due to the persistence of fever, an additional course of empiric broad-spectrum antibiotic therapy with meropenem 2 g t.i.d. was started, and a myeloid activation test at flow cytometry on peripheral blood was performed, which showed a picture of marked granulocytic activation (i.e., a granulocyte/lymphocyte ratio of CD64 intensity expression: 13.8; normal value < 2.6) consistent with a possible infection. Therefore, an antifungal therapy (caspofungin 70 mg on day 1 and 50 mg q.d. for 3 days) was added to meropenem, again without any improvement. Because of all these treatment failures, after a multidisciplinary discussion, it was finally decided a steroid therapy with methylprednisolone 125 mg t.i.d. would be started due to suspicion of an autoimmune disease. For the differential diagnosis between infectious and autoimmune diseases, the patient underwent all of the additional investigations reported in Table S4 in the meantime.

**Figure 2:**
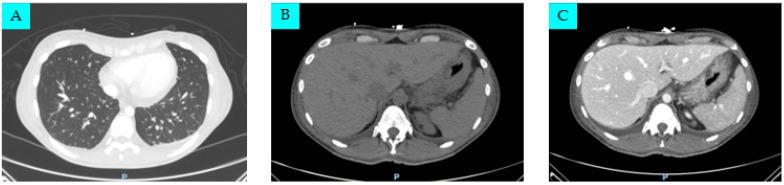
Patient radiological aspect in total body computed tomography (CT) scan: (**A**) chest CT revealing a 22 mm pericardial effusion and bilateral pleural effusions; (**B**) abdomen CT revealing severe hepatomegaly and moderate splenomegaly (before contrast); (**C**) another image of the same abdominal aspect (portal venous phase).

Total body computed tomography was also performed, showing a 22 mm pericardial effusion, bilateral pleural effusions, and a pelvic intraperitoneal fluid flap; both the liver and spleen were enlarged and a small amount of free fluid was reported within the peritoneal space and the paracolic gutters and around the liver and gallbladder (Figure 2).

**Figure 3:**
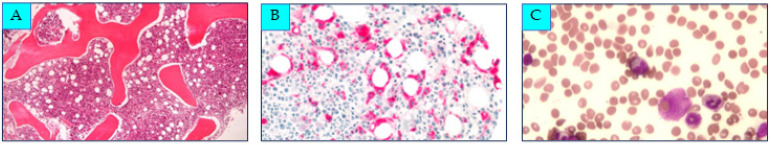
Patient bone marrow biopsy and aspiration showing hemophagocytic syndrome. (**A**) Bone marrow is highly hypercellular and shows predominance of mature myeloid elements; megacariocytes are well represented; cells of eritroblastic lineage are heavily reduced (hematoxylin–eosin, 100×); (**B**) macrophages were very numerous and collected in small aggregates or surrounding the adipocytes (CD68 immunostaining, 200×); (**C**) on smears of bone marrow, evidence of macrophages phagocytizing erythrocytes was observed (May-Grṻnwald Giemsa, 300×).

Due to the occurrence of anemia and thrombocytopenia, requiring red blood cell/plasma transfusions and human fibrinogen concentrate administrations, a diagnosis of acute disseminated intravascular coagulation was made; prothrombin time was moderately prolonged, while activated partial thromboplastin time, ADAMTS13 enzyme activity levels, and schistocyte count at peripheral blood smear were within normal ranges. Autoantibodies directed against ADAMTS13 were absent, and the Coombs test (direct and indirect) was negative. Flow cytometry analysis of bone marrow/peripheral blood did not show any clonal B-cell or abnormal T-cell populations, while it did confirm inflammatory circulating neutrophilic leukocytosis. A bone marrow biopsy was finally performed, revealing the presence hemophagocytosis on the bone marrow smear and a histological pattern of hypercellular marrow with mature myeloid hyperplasia, a marked reduction/absence of erythroblastic series, and diffuse infiltration of histocytes, some of which had a hemophagocytic pattern (Figure 3).

After the procedure, due to the appearance of desaturation (arterial partial pressure of oxygen in room air: 44.0 mmHg) and orthopnea, it was necessary to put the patient on a course of continuous positive airway pressure (CPAP), which was later discontinued due to the improvement in respiratory failure.

Both the aforementioned biopsy findings and the appearance of a weak clinical response only after the initiation of steroid therapy guided, at this stage, the diagnostic suspicion toward the presence of an HLH, possibly secondary to an autoimmune disease. In this respect, the onset of AOSD appeared to be plausible, so Yamaguchi’s classification criteria were applied (Table S1) [3]. A positivity for all four major and all five minor criteria was found. The diagnosis was also supported when applying the Fautrel criteria (Table S2); for this, five out of the six major criteria were met (the only criterion that was not confirmed was glycosylated ferritin, simply because it was not tested). Also, both the minor criteria were fulfilled [4]. This is despite the possible concomitant infectious process, which in itself would be an exclusion criterion according to Yamaguchi’s classification; in any case, infections alone—even had they been present—could not have accounted for the patient’s clinical and histological patterns. Therefore, a final diagnosis of MAS as a complication of new-onset AOSD was finally made.

Given the patient’s progressive and rapid deterioration, treatment with an intensive therapy with immunosuppressive and cytotoxic agents was administered with the aim to induce remission of the disease activity, following the HLH-94 treatment protocol [13]. More in detail, this initial therapy included etoposide, which is proapoptotic, in HLH-150 mg/m^2^ IV twice weekly during the first 2 weeks and then weekly, in combination with daily dexamethasone (initially 10 mg/m^2^ IV for 2 weeks, followed by 5 mg/m^2^ IV for 2 weeks, and 2.5 mg/m^2^ IV for 2 weeks). For the common side effect of etoposide-induced neutropenia, filgrastim was introduced, while trimethoprim/sulfamethoxazole (180/800 mg/q.d. orally) and acyclovir (800 mg/q.d. intravenously) therapy were started as secondary prophylaxis.

After the first two doses of chemotherapy administered in the ICU, the patient became hemodynamically stable again and could be transferred to an Internal Medicine Division, where she experienced progressive rapid clinical and laboratory improvement. More in detail, the fever completely resolved, and the performance status significantly recovered, although some fatigue remained. From a musculoskeletal perspective, arthralgias gradually reduced until they disappeared, but a marked sarcopenia, requiring an in-hospital rehabilitation program, persisted as a result of the prolonged intrahospital bed rest. With regard to the main laboratory issues, the inflammatory indices gradually decreased to normalization, platelet and hemoglobin levels stabilized, and transaminase and ferritin levels dramatically reduced (Table 1).

Two months after the initial hospitalization the patient was finally discharged in stable clinical condition (switching dexamethasone to oral prednisone at an initial dosage of 25 mg q.d.) and referred to our rheumatology outpatient clinic. During the first eight follow-up months, she remained apyretic and symptom-free at home except for a residual easy fatigability and persistent arthralgias. From a biochemical point of view, the only relevant data consisted of a slight transient increase in lactate dehydrogenase (2.0 × upper limit of normal, ULN) and transaminase levels (1.7× ULN) two weeks after hospital discharge. This pattern was interpreted as the tail end of MAS or secondary to AOSD, in which modest elevations of serum hepatic liver enzymes are very common and generally related to the disease, rather than drugs used to treat AOSD, so that they improve as the disease responds [17]. Methotrexate (MTX) 12.5 mg SC quaque week was then chosen, despite the lack of normalization of the transaminases, because of its known efficacy in addressing AOSD arthritic manifestations, while acting as a glucocorticoid-sparing agent [18,19]. The drug was well tolerated, as confirmed by laboratory tests that were completely normal within the second month of follow-up and from there on. In the meantime, the patient continued her slow tapering of the steroid therapy (current prednisone dosage: 2.5 mg q.d.) (Table 1).

## 3. Discussion

This case highlights the difficulty in managing a young and previously healthy individual who rapidly deteriorated and developed life-threatening conditions. She initially presented mild symptoms, interpreted as a routine bacterial upper respiratory tract infection. However, subsequently, the clinical course changed, and the following impression was of an infection of unknown origin in the septic evolution phase. And it is on this front that the doctors’ attention was focused during the first part of the hospitalization, being influenced by the aforementioned initial misleading elements. The key challenge in this clinical case was really distinguishing between infection and autoimmunity. Importantly, both conditions can coexist, and an infection may even trigger autoimmunity. Moreover, immune activation from infection is also a common trigger for MAS-HLH both in patients with a genetic predisposition and in sporadic cases with no underlying genetic cause identified.

Our patient was ultimately affected by AOSD. This is a rare systemic autoinflammatory disorder characterized by recurrent high fever, a fading salmon-pink rash, and arthritis. These signs/symptoms are often associated with pharyngodynia, myalgias, lymphadenopathy, splenomegaly, and neutrophilic leukocytosis (Table S5) [17,20,21,22,23,24,25,26,27,28,29,30,31].

In young individuals, AOSD’s counterpart is called systemic juvenile idiopathic arthritis (s-JIA), with shared symptoms like recurrent fever, rash, and polyarthritis [1]. This type of manifestation is shared by various pathologies that are much more common than AOSD; in fact, in our case, they were initially interpreted as being of bacterial or fungal origin. The etiology of the disease remains unknown, but it is believed that various infectious agents may trigger it in susceptible individuals, such as Rubivirus, Morbillivirus, Mycoplasma Pneumoniae, or Chlamydia Pneumoniae [32]. However, no single pathogenic trigger has been clearly identified, suggesting the involvement of multiple factors [33]. In our young patient, no definitive elements pointed to a specific causative agent.

Being a rare disease, AOSD may be difficult to diagnose. This condition exhibits common characteristics with lymphoma, solid tumors, and infections, including unusual ones, particularly in the case of young patients. In this particular case, the presence of normal procalcitonin values, despite high C-reactive (CRP) protein levels, and the absence of a response to proper antibiotic therapy suggested a non-bacterial origin for the underlying condition. Additionally, there could have been several nuances that further complicated the differential diagnosis, including the presence of elevated serum ferritin levels—which also generally contribute to differentiating between infectious and noninfectious diseases in patients with fever of unknown origin—and polyserositis, an uncommon manifestation of an already rare disease [34].

Patients typically face a journey of confusing symptoms, misdiagnoses or delayed diagnoses, and a series of ineffective treatments before a proper diagnosis and an effective treatment plan can be provided. All this can lead to longer hospital stays and higher financial costs, but it can also hasten the onset of the rare and potentially fatal AOSD complications that we will discuss below. In any case, a diagnosis is generally made by exclusion, as clinicians do not currently have biomarkers to make a definitive diagnosis and must rely on a few specific sets of diagnostic criteria, among which the most sensitive and widely used are the Yamaguchi ones, as shown in Table S1 [3]. A diagnosis can be made when ≧5 criteria are present, with at least 2 being major diagnostic criteria, preferably in the absence of any exclusion criterion. The Fautrel classification, the other set of criteria which has been validated, has the advantage of including ferritin and glycosylated ferritin levels as diagnostic biomarkers and does not require exclusion criteria; in this case, diagnosis requires four or more major criteria or three major criteria plus two minor criteria (Table S2) [4]. Despite their widespread use and proven clinical utility, both these criteria only consider the patient clinical presentation, which is sometimes nuanced and, in rare cases, overlapping with a transient acute infectious pattern that may facilitate the onset of AOSD itself, as reported above [35], but at the same time, infection may lead to misdiagnosis and complicate clinical outcomes.

It is important to note that AOSD is a complex and multifaceted condition. It is generally a mild disease, but approximately 20% of patients develop a potentially fatal complication, the most frequent being MAS (also called reactive hemophagocytic syndrome), a secondary form of HLH, differing from the latter one primarily through more pronounced involvement of the myocardium and coagulation but coupled with fewer cytopenic manifestations. More in detail, MAS can manifest with rash, fever, pancytopenia, hepatosplenomegaly, and systemic involvement, leading to death in about 10% of cases. This is because it is associated with a cytokine storm and overactive monocytes/macrophages, causing multi-organ dysfunction [36,37]. For mild AOSD cases, NSAIDs alone may be sufficient, but moderate-to-severe cases often require initial CS. However, continuous steroid use has significant toxicities, so CS-sparing drugs like MTX, cyclosporine A (CSA), and leflunomide are commonly used for achieving steroid-free remissions and managing refractory cases [36]. In addition, recently developed biologic drugs have been proposed for the most severe and refractory subjects where they represent an effective and reliable alternative to conventional synthetic disease-modifying antirheumatic drugs (csDMARDs) as well as the only remaining treatment option. Currently, a safe and effective strategy is the specific suppression of IL-6 (with tocilizumab) or IL-1 (with anakinra, canakinumab, or rilonacept). Tumor necrosis factor (TNF) inhibitors (such as infliximab, etanercept, or adalimumab) and IVIG may be used in refractory arthritis forms [38], while B-cell depletors (rituximab) have been reported as effective in AOSD complicated by thrombotic microangiopathy [39].

For patients at risk of MAS-HLH, a personalized and graded treatment approach is advised. Generally, a combination of pulse-dose IV CS and high-dose anti-IL-1 anakinra is suggested [15]. MAS suspicion is particularly high with elevated ferritin levels disproportionate to other inflammatory markers, transaminase elevation, marked D-dimer elevation, thrombocytopenia, and/or decreasing erythrocyte sedimentation rate despite continued CRP elevation [40]. In the case herein described, an MAS-HLH diagnosis was not only suspected but also confirmed, meeting seven out of nine HLH-2004 criteria, in addition to the finding of increased liver enzymes (Table S3); moreover, the H score was 215, indicating an over 95% probability of HLH [8,10,11,41]. For confirmed or strongly suspected MAS, current guidelines recommend empiric treatment for the presumed underlying pathology, deferring HLH-specific therapy unless vital functions deteriorate. Instead, acutely ill patients, as in the present case, should be promptly treated. In addition to the treatments already mentioned (CS + anakinra), CSA (despite its safety profile problems) as well as IL-6-blocking therapy with tocilizumab may be considered, particularly in patients with an insufficient immediate response. In this setting, the HLH-94 protocol—originally validated for primary (genetic) HLH forms—also remains one of the recommended standards of care [15]. It involves etoposide and dexamethasone and grants a median survival of 54% at 6.2 years [13]. While similar alternative regimens do exist, like the HLH-2004 protocol, none has shown clear superiority. Finally, clinicians should also consider including patients in clinical trials, when available.

As mentioned, etoposide, a commonly prescribed anticancer drug, is part of the HLH-94 protocol. This drug acts by inhibiting DNA topoisomerase II and inducing permanent DNA breaks and cell cycle inhibition, so its prolonged use is associated with therapy-induced secondary leukemia, especially in children. It is now increasingly being used also in non-tumor contexts, such as immune-mediated inflammatory disorders like MAS-HLH, due to its activity in dampening inflammation and inhibiting pro-inflammatory cytokine production [42,43]. In general, the action of etoposide decreases the direct activity of T lymphocytes and globally suppresses the cytokine cascade (with particular regard to IL-6, IL-10, IL-18, interferon (IFN)-γ, and TNF-α), facilitating the cessation of the MAS-HLH process. This mechanism engages various components that are crucial for the proper functioning of the immune system, also resulting in some transient increased risk of opportunistic infections which is difficult to quantify precisely. However, it is plausible that interrupting the cytokine cascade involves numerous molecules and may potentially have a more significant impact on the immune system than blocking a single cytokine, such as IL-1 or IL-6. Unfortunately, head-to-head studies or at least indirect comparisons are—to the best of our knowledge—not currently available, so it is correct to always initiate infection prophylaxis if etoposide is used, as was done in the present study.

The decision to follow the HLH-94 scheme in this specific, very serious case was influenced by the medical team’s familiarity with the protocol and the collaborative management by internists, rheumatologists, and hematologists. In any case, whichever schedule is chosen, it is important to bear in mind that the management of MAS-HLH should ideally always involve a multidisciplinary approach; as a matter of fact, beyond merely following the recommendations outlined in the literature, the incorporation of diverse perspectives enhances competence and improves the likelihood of achieving a favorable outcome [15,44,45].

Finally, it should be considered that the original HLH-94 and HLH-2004 protocols were developed for pediatric patients with primary HLH, and their application in adults, especially those with AOSD-MAS, may lead to overtreatment and increased toxicity [13,15]. Thus, dose reduction and personalized treatment are recommended [15,46,47]. However, there is a lack of guidance on the proper application of such protocols for these subjects. For instance, a recent study suggested that modifying the HLH-2004 scheme with low-dose, short time courses of etoposide showed improved survival rates and clinical outcomes, together with a better safety profile and reduced CS exposure [48]. Based on these considerations, in the case herein described, the HLH-94 classical regimen was also modified by the multidisciplinary team, in that the patient’s initial clinical response was so dramatically good that the etoposide induction phase could be shortened to only 5 weeks instead of the 8 weeks described in the original protocol, while continuing slow CS tapering (Table 1).

## 4. Conclusions

AOSD is a rare autoinflammatory disease with diverse clinical presentations and non-specific features that can mimic various conditions. MAS-HLH, a life-threatening complication of AOSD, occurs in a significant proportion of patients, with a short-term mortality rate as high as 10% [48]. Diagnosis for both conditions can be prolonged, especially when confounding factors are present. In the described case, the initial suspicion of systemic infection and iatrogenic Clostridium Difficile colitis initially ruled out AOSD, despite the diagnostic criteria being fulfilled. AOSD was considered only when HLH developed, leading to the hypothesis of MAS as a complication triggered by an infectious/inflammatory background. Timely initiation of high-dose steroids and topoisomerase II inhibitors proved crucial and effective.

Despite the increased understanding of AOSD, it remains a diagnostic and therapeutic challenge with many gaps in knowledge, especially concerning AOSD-MAS. Current standards of care, such as, for instance, the HLH-94 protocol, have improved survival, but mortality remains high. Additionally, applying pediatric criteria and treatments to adults may not always be suitable.

This research highlights the importance of a multidisciplinary team (including, in the present case, internists, rheumatologists, immunologists, pathologists, hematologists, radiologists, and infectious disease and critical care medicine specialists) for the survival and recovery of patients when facing serious, life-threatening complications.

## Figures and Tables

**Table 1:** Main patient laboratory findings. Bold values are outside local laboratory normal ranges.

	Admission	VP-16 1st Dose	VP-16 2nd Dose	VP-16 3rd Dose	Vp-16 4th Dose	VP-16 5th Dose	Vp-16 6th Dose	VP-16 7th Dose	Discharge	f/up 2 mos	f/up 8 mos	Local Laboratory NR
WBCs	**10.84**	**12.97**	**3.05**	**2.99**	**2.36**	**6.65**	**3.91**	**9.16**	**4.55**	6.64	7.76	4.50–11.00 × 10^9^/L
Neutrophils	**9.42**	**10.99**	2.59	2.42	**1.54**	3.18	**1.61**	5.31	2.24	4.03	4.85	1.80–7.70 × 10^9^/L
Lymphocytes	1.00	**0.67**	**0.39**	**0.51**	**0.81**	2.53	2.02	2.96	1.83	1.93	2.20	1.00–4.50 × 10^9^/L
Hb	**9.2**	**7.9**	**8.9**	**8.7**	**9**	**8.5**	**8.2**	**8.1**	**8.5**	12.2	**11.0**	11.5–15.5 g/dL
PLTs	296	**30**	**36**	**46**	**56**	**76**	238	176	152	197	191	130–400 × 10^9^/L
INR	**1.38**	**1.75**	**1.64**	**1.50**	**1.47**	1.19	1.09	1.05	1.02	1.01	1.01	0.8–1.2 Units
Fibrinogen	**112**	**96**	**98**	**85**	**83**	**107**	**66**	**137**	209	/	/	200–393 mg/dL
Glycemia	**158**	**183**	**162**	88	76	70	**106**	73	79	80	78	74–100 mg/dL
Creatinine	0.72	0.55	0.37	0.22	0.40	0.49	0.47	0.42	0.44	0.65	0.65	0.5–121 mg/dL
Na	136	137	137	135	136	137	136	139	141	143	144	134–146 mmol/L
K	3.5	4.4	4.0	4.2	4.1	4.1	4.0	4.0	4.0	3.8	4.0	3.4–4.5 mmol/L
Triglycerides	**309**	/	/	**226**	64	/	/	/	/	/	/	<150 mg/dL
CRP	**11.38**	**2.10**	**1.04**	0.32	0.22	/	/	0.12	/	0.03	0.02	0–0.50 mg/dL
PCT	0.3	0.2	0.3	<0.05	/	/	/	/	/	/	<0.05	<0.5 µg/L
Ferritin	**1059**	**1608**	**1338**	**553**	**574**	**628**	**531**	**580**	**555**	19	38	13–150 µg/L
LDH	**970**	**1727**	**735**	**562**	**530**	**461**	**475**	**568**	/	443	27	208–450 U/L
Total bilirubin	0.55	**1.63**	**1.58**	**1.24**	**1.36**	**1.43**	1.00	0.66	1.05	0.70	0.81	0.30–1.20 mg/dL
AST	**49**	**590**	**100**	39	27	21	27	30	28	24	19	0–40 U/L
ALT	**42**	**620**	**349**	**188**	**127**	**55**	**56**	**62**	**61**	15	17	0–40 U/L
GGT	**74**	**199**	**288**	**239**	**177**	**93**	**79**	**85**	**85**	26	34	0–50 U/L
ALP	**187**	**139**	104	87	79	66	61	58	66	**31**	70	46–116 U/L

ALP: alkaline phosphatase; ALT: alanine transaminase; AST: aspartate transaminase; CRP: C-reactive protein; GGT: gamma-glutamyl transferase; f/up: follow-up; Hb: hemoglobin; INR: international normalized ratio; K: potassium; LDH: lactate dehydrogenase; mos: months; Na: sodium; NR: normal range; PCT: procalcitonin; PLTs: platelets; VP-16: etoposide; WBCs: white blood cells; /: not tested.

## Data Availability

All data generated or analyzed during this study are included in this published article and its supplementary information files.

## Supplementary Materials

The following supporting information can be downloaded at: https://www.mdpi.com/article/10.3390/life14020195/s1. Table S1: Yamaguchi criteria for diagnosing adult-onset Still’s disease (AOSD); Table S2: Fautrel criteria for diagnosing adult-onset Still’s disease (AOSD); Table S3: main diagnostic (clinical, laboratory, and histopathologic) criteria for hemophagocytic lymphohistocytosis (HLH); Table S4: results of other laboratory analyses performed during hospitalization; Table S5: characteristics of the clinical manifestations of adult-onset Still’s disease (AOSD).
