# Supplementary material for: Unveiling the Mystery of Adult-Onset Still’s Disease: A Compelling Case Report
Source: Life (Basel). 2024 Jan 29;14(2):195. doi: 10.3390/life14020195 (PMC10890189; doi:10.3390/life14020195)
Supplement: Supplementary file 1 [file life-14-00195-s001.zip › life-2824248-supplementary.pdf]

life

Case Report

Unveiling the Mystery of Adult-Onset Still's Disease: A Compelling Case Report

Daniele Sola [1,2,3,4,*,†], Carlo Smirne [1,2,†], Francesco Bruggi [1,2], Chiara Bottino Sbaratta [1,2], Aubin Cardin Tamen Njata [1,2], Guido Valente [1,5], Maria Cristina Pavanelli [5], Rosetta Vitetta [6], Mattia Bellan [1,2,3,4], Lorenzo De Paoli [7] and Mario Pirisi [1,2,3,4]

Table S1. Yamaguchi criteria for diagnosing adult-onset Still's disease (AOSD) [3].

**Major criteria**
Fever ≥ 39 °C, lasting ≥ 1 week
Arthralgias or arthritis lasting ≥ 2 weeks
Typical salmon-pink nonpruritic skin rash
Peripheral blood leukocytosis ≥ 10,000/µL, with granulocytes ≥ 80%
**Minor criteria**
Pharyngodynia
Lymphadenopathy
Hepatomegaly and/or splenomegaly
Abnormal liver function tests
Negative tests for rheumatoid factor (RF) and antinuclear antibodies (ANAs)
**Suggested exclusion criteria:**
Infections (especially sepsis and infectious mononucleosis)
Malignancies (especially malignant lymphoma)
Other rheumatic diseases (especially polyarteritis nodosa and rheumatoid vasculitis with extra-articular features)

Table S2. Fautrel criteria for diagnosing adult-onset Still's disease (AOSD) [3].

**Major criteria**
Spiking fever ≥ 39 °C
Arthralgia
Transient erythema
Pharyngitis
Polymorphonuclear leukocytes (PMNs) ≥ 80%
Glycosylated ferritin ≤ 20%
**Minor criteria**
Maculopapular rash
Leukocytes ≥10,000/µL
**Suggested exclusion criteria**
None

Table S3. Main diagnostic (clinical, laboratory, and histopathologic) criteria for hemophagocytic lymphohistocytosis (HLH) [8,10]. Diagnosis is generally made when 5 of the following 9 criteria used in the HLH-2004 trial are met, but some authors consider the following modified criteria sufficient: 3 of 4 clinical findings (HLH-2004 criteria 1 to 3 and hepatitis) plus abnormality of 1 of 4 immune markers (HLH-2004 criteria 5 to 8) [1] [10,11].

1. Fever ≥ 38.5 °C, lasting ≥1 week
2. Splenomegaly (≥3 cm from the costal arch)
3. Cytopenias (≥2 of 3 hematopoietic lines involved in the absence of myelodysplasia) [2]

4. Hypertriglyceridemia [3]
5. Hypofibrinogemia [4]
6. Hemophagocytosis on specimens from bone marrow, spleen, or lymph nodes in the absence of malignancy
7. Ferritin >500 μg/L [5]
8. Low/absent NK cell activity [6]
9. Soluble CD25 (Interleukin-2 receptor alpha) and/or CXCL9 elevation

[1] Further laboratory and radiographic abnormalities in other organ systems not used in the HLH-2004 trial may include liver function and coagulation abnormalities (including bleeding manifestations); neurologic abnormalities (such as seizures, mental status changes, and ataxia); respiratory abnormalities (including acute respiratory distress syndrome); severe hypotension requiring the administration of one or more vasopressors; renal dysfunction with or without hyponatremia (including renal failure requiring dialysis); skin manifestations (including rashes, erythroderma, edema, petechiae, and purpura); clinical features of Kawasaki disease (including conjunctivitis, red lips, and cervical lymphadenopathy) [9]. [2] Hemoglobin <9 g/dL; platelets $<100 \times 10^9$/L; absolute neutrophil count $<1.0 \times 10^9$/L. [3] Fasting triglycerides > 265 mg/dL. [4] Considered at < 150 mg/dL. [5] > Considered at 500 ng/mL (but ferritin > 3000 ng/mL has a better specificity and positive predictive value). [6] Two standard deviations above age-adjusted, laboratory-specific normal values.

Table S4. Results of other laboratory analyses performed during hospitalization. Bold values are outside local laboratory normal ranges.

Laboratory test	Result	Local Laboratory NR
**C3/C4**	1.45/0.19	0.90–1.80/0.10–0.40 g/L
**Rheumatoid factor (RF)**	negative	-
**ANAs**	negative	-
**dsDNA Abs**	9.5	0–27 IU/ml
**Anti-ENA antibody screen** [1]	<3.6	0–20 CU
**Autoimmune liver disease panel** [2]	0	0–6 CU
**ANCAs**	negative	-
**LA testing, SCT screening ratio**	0.94	0.77–1.20
**LA testing, dRVTT screening ratio**	1.06	0.70–1.20
**Anti-cardiolipin IgG**	0.7	0–20 CU
**Anti-cardiolipin IgM**	7.5	0–20 CU
**Anti-beta2glycoprotein IgG**	3.9	0–20 CU
**Anti-beta2glycoprotein IgM**	1.8	0–20 CU
**Beta-2 microglobulin**	2.40	1.16–2.52 mg/L
**Serum IgG**	806	751–1560 mg/dL
**Serum IgM**	**222**	48–220 mg/dL
**Serum IgA**	182	80–400 mg/dL
**Quantiferon-TB Gold Plus test** [3]	negative	<0.35 UI/mL
**HIV Ab**	negative	-
**HBsAg**	negative	-
**HCV Ab**	negative	-
**ASLO**	45	<200 IU/mL
**Anti-Parvovirus B19 IgG, index**	**2.0**	0.90–1.20
**Anti-Parvovirus B19 IgM, index**	**<0.10**	0.90–1.10
**Widal–Wright** [4]	< 1/80	<1/80
**Fecal calprotectin**	6.77	<50 μg/g
**SARS-CoV-2 antigen rapid test** [5]	negative	-
**Serum Aspergillus antigen, ratio**	0.10	0.00–0.16
**CMV DNA**	negative	-
**EBV DNA**	negative	-

**Interleukin-6**	2.7	0.0–4.4 pg/mL
**Anti-tTG IgA**	1.7	0.0–20.0 CU
**TSH**	1.08	0.36–3.74 mIU/L
**FT4/FT3**	8.0/1.2	9.0–17.0/2.7-4.4 ng/L
**TG Ab/TPO Ab**	30/12	10-115/0-35 IU/mL

Ab: antibodies; ANAs: antinuclear antibodies; ANCAs: antineutrophil cytoplasmic antibodies; ASLO: antibodies anti-streptolysin O; CMV: Cytomegalovirus; C3: complement component 3; C4: complement component 4; CU: chemiluminescent units; dRVVT: dilute Russell's viper venom time; dsDNA Abs: anti-double-stranded deoxyribonucleic acid antibodies; EBV: Epstein–Barr virus; ENA: extractable nuclear antigen; FT4: thyroxine; FT3: triiodothyronine; IU: international units; HBsAg: hepatitis B surface antigen; HCV: hepatitis C virus; HIV: human immunodeficiency virus; Ig: immunoglobulin; LA: lupus anticoagulant; NR: normal range; SARS-CoV-2: severe acute respiratory syndrome coronavirus 2; SCT: silica clotting time; TB: tuberculosis; TG: thyroglobulin; TPO: tireoperoxidase; TSH: thyroid-stimulating hormone; tTG: tissue transglutaminase. [1] Including the following autoantibodies: anti-Sm (anti-Smith), anti-RNP (anti-ribonucleoprotein), anti-SSA (anti-Sjögren's syndrome type A)/Ro60, anti-SSA/Ro52, anti-SSB (anti-Sjögren's syndrome type B), anti-Jo-1 (anti-histidyl tRNA synthetase), and anti-Scl 70 (anti-topoisomerase I). [2] Including the following autoantibodies: anti-LKM1s (antibodies to liver/kidney microsome type 1); ASMAs (anti-smooth muscle antibodies), AMAs (anti-mitochondrial antibodies), anti-LC1 (anti-liver cytosolic antigen type 1), anti-SLA (anti-soluble liver antigen), anti-gp210 (anti-glycoprotein210), anti-SP100 (anti-SP100 nuclear antigen). [3] interferon gamma dosage. [4] Including the following antibodies: anti-Typhus; anti-Paratyphoid; anti-Brucella. [5] Nasal swab.

Table S5. Characteristics of the clinical manifestations of adult-onset Still's disease (AOSD).

**Fever** [20]	Fever is almost always daily, with no spontaneous intercritical intervals. Very characteristically, it is biphasic (2 peaks within the same day) with a sudden temperature increase (4 °C in 4 h).
**Rash** [21]	The rash is asymptomatic concerning itching or pain, occurs together with fever, and disappears when the temperature returns to normal (fleeting). It presents as spots or maculopapules, usually on the trunk and extremities, rarely on the palmoplantar areas or on the face. The Koebner phenomenon (more intense in the areas of stress from the clothes) is frequently observed.
**Arthritis** [22]	Arthritis is typically transient and mild (however, cases of severe synovitis leading to joint destruction have been described). It is a migrating oligoarticular manifestation, with a preference of, in order of probability: knees, wrists, metacarpophalangeal/proximal interphalangeal joints, ankles, elbows, and shoulders.
**Myalgia** [17]	Myalgia is closely related to fever; the disease does not specifically attack the muscles; in fact, electromyography and muscle biopsies in these cases are always normal. However, the dosage of muscle enzymes in the acute phases of the disease can show increases, albeit slight.
**Pharyngitis** [23]	Sore throat is a characteristic of the disease, especially at onset before the occurrence of fever, and should always be sought in the patient's anamnesis when there is a suspected diagnosis. It is a non-suppurative cricothyroid perichondritis or aseptic non-exudative pharyngitis of a purely inflammatory nature.
**Lymphadenopathy** [24]	Lymphadenopathy affects more than 2 out of 3 people with AOSD. It is typically a symmetrical lymphadenopathy involving neck lymph nodes, which feel soft or stretchy to the touch. This phenomenon is due to benign B-cell hyperplasia of the pericortical zone.
**Splenomegaly** [24]	Acute splenomegaly is present in at least 33% of patients at diagnosis. It typically occurs in the absence of clinical hyperplenism, and it is not painful.
**Liver disease** [24]	Elevated liver necrosis rates are more frequent than hepatomegaly. In general, these are transient and non-dangerous conditions, even if cases in which fulminant hepatitis develops (all in patients treated with high doses of NSAIDs) have been reported.

**Cardiac and pulmonary disease** [25,26]	Cardiac (non-ischemic) involvements including arrhythmias and pericarditis have been described, as well as pulmonary infiltrates and pleural effusions, which together do not represent a significant proportion of patients but should be considered possible and should not confuse the physician in the differential diagnosis.
**Hematologic manifestations** [27]	Microangiopathic hemolytic anemia, hemolytic uremic syndrome, and thrombotic thrombocytopenic purpura have all occasionally been described, in addition to the fearsome MAS.
**Gastrointestinal symptoms** [24,28]	The presence of abdominal pain is highly variable in AOSD, generally in relation to the onset of fever; however, there are reports of pancreatitis and aseptic peritonitis.
**Others** [24,29–31]	Rare manifestations include conjunctivitis, uveitis, aseptic meningitis, interstitial nephritis, glomerulonephritis, and secondary amyloidosis.

AOSD: adult-onset Still's disease; MAS: macrophage activation syndrome; NSAIDs: non-steroidal anti-inflammatory drugs
